# Heat at Mosul

**Published:** 1846-11

**Authors:** 


### SELECTIONS

FROM

### AMERICAN AND FOREIGN JOURNALS.

---

*Heat at Mosul.*—Azariah Smith, M.D., in the missionary service, has published, in the American Journal, an interesting series of observations on the meterology of Western Asia. He states that the mercury in the thermometer, placed in the sun at noon, would always rise to 144° or 146° at Mosul, near the supposed ruins of ancient Nineveh. Such of the inhabitants as are able, have rooms fitted up in their cellars, where they retreat in the middle of the day. The nights are uniformly spent on the roof—dew and rain being wholly unknown during the summer season. Contact with anything dry, communicates the sensation of heat. The beds, says Dr. Smith, seemed to have been just scorched with a warming pan; and stone floors appeared as if endowed with the power of generating caloric. Instead of being refreshed by the cooling sensation which a change of clothes ordinarily gives in the summer, the linen taken out of our coolest wardrobes seemed always, on putting it on, to have come roasting hot from the mouth of some glowing furnace.—*Boston Med. and Surg. Jour.*

